# Examining the Gaps and Issues of End-of-Life Care among Older Population through the Lens of Socioecological Model—A Multi-Method Qualitative Study of Hong Kong

**DOI:** 10.3390/ijerph17145072

**Published:** 2020-07-14

**Authors:** Roger Yat-Nork Chung, Dong Dong, Nancy Nam Sze Chau, Patsy Yuen-Kwan Chau, Eng Kiong Yeoh, Eliza Lai-Yi Wong

**Affiliations:** School of Public Health and Primary Care, The Chinese University of Hong Kong, Hong Kong, China; dongdong@cuhk.edu.hk (D.D.); nancy.chau@cuhk.edu.hk (N.N.S.C.); patsychau@cuhk.edu.hk (P.Y.-K.C.); yeoh_ek@cuhk.edu.hk (E.K.Y.); lywong@cuhk.edu.hk (E.L.-Y.W.)

**Keywords:** end-of-life care, palliative care, older persons, Hong Kong, Chinese

## Abstract

End-of-life (EOL) care for terminal illness and life-limiting conditions is a sector in the health service spectrum that is drawing increased attention. Despite having the world’s longest life expectancy and an ever-escalating demand for long-term care, Hong Kong’s EOL care was underdeveloped. The current study aims to provide a holistic picture of gaps and issues to EOL care in Hong Kong. Data collection was conducted using a multi-method qualitative approach that included focus groups and in-depth interviews with key informants and stakeholders, and longitudinal case studies with patients and families. Deductive thematic analysis was used to examine service gaps in current EOL care through the lens of a socioecological model where gaps and issues in various nested, hierarchical levels of care as well as the relationships between these levels were studied in detail. Using the model, we identified gaps and issues of EOL care among older populations in Hong Kong at the policy, legal, community, institutional, as well as intrapersonal and interpersonal levels. These include but are not limited to a lack of overarching EOL care policy framework, ambiguity in the legal basis for mental incapacity, legislative barriers for advance directives, inadequate capacity, resources, and support in the community to administer EOL care, inadequate knowledge, training, and resources for EOL care in health and social care sectors, inadequate medical-social interface, general reluctance and fear of death and dying, as well as the cultural interpretation of filial piety that may lengthen the suffering of the dying patients. Findings highlight the multi-level gaps and issues of EOL care in a place where western and eastern culture meet, and shed light on how best to design more effective and comprehensive policy interventions that will likely have a more sustainable and instrumental impact on facilitating person-centered EOL care during the end of life.

## 1. Introduction

End-of-life (EOL) care, instead of being a standalone type of care, is an integral part of the long-term care that specifically regards the care of the last moment of life. While the quality of life at the end matters for everyone, death and dying have long been and still are taboo subjects that are difficult to be openly discussed in many cultures [1,2,3,4]. It was not until recent years that public engagement and policy interventions with the aims to improve quality of death through provision of high-quality EOL care have gained momentum, as “dying a good death” becomes more discussed and deliberated in the community. Albeit a topic of constant debate, the concept of “a good death” often relates to being better prepared when death is approaching, and minimizing suffering for patients and their families, while maximizing dignity, privacy, and self-control over EOL care plan and place of deaths [5,6].

In more recent years, the provision of quality EOL care has been increasingly recognized. For instance, the World Health Organization [7] in its illness trajectory framework highlighted that palliative care should be increasingly administered according to the patient’s need as the disease progresses, not only at the very end of life; the National Institute for Health and Care Excellence of the UK developed clinical guidance specifically for Care of Dying Adults in the Last Days of Life [8] in 2015; and the Institute of Medicine (now National Academy of Medicine) of the US also published Dying in America: Improving quality and honoring individual preferences near the end of life [9]. Despite the increasing recognition, even to this day, many clinicians and healthcare professionals still view palliative care as a sign of giving up hope, and are thus reluctant to recommend it to their patients [4]. Some also believe that referral to palliative services will shorten life due to withdrawal or withholding of life-sustaining treatments. However, a randomized controlled trial showed that cancer patients who received palliative care along with standard curative care had better quality of life and longer life span than those who received only standard curative care [10], while another trial also found that integrated palliative and respiratory care for patients with advanced disease and refractory breathlessness also improved survival for patients with chronic obstructive pulmonary disease and noncancerous lung disease [11].

Hong Kong, a former British colony and now a special administrative region of China with more than 7 million people where the cultures of the East and West meet, has the longest average life expectancy at birth in the world, even surpassing that of the traditional champion of the indicator, Japan [12]. Yet according to the Economist Intelligence Unit’s Quality of Death Index, an instrument used to highlight the advances made, as well as the challenges and gaps in policy and infrastructure of EOL care of 80 countries around the world [13], Hong Kong was ranked relatively low at number 22 in the overall score, behind other developed Asian economies, including Taiwan, Singapore, Japan and South Korea, as well as its former sovereignty state, the UK, which ranks number one. Although Hong Kong’s tax-based public healthcare system was based on that of the UK National Health Service and many of the aspects of lifestyle are influenced by its former colonist, the situations of EOL care are quite different. Over 90% of deaths in Hong Kong occurred in hospitals [14], as opposed to 48.5% in the UK [15]. Although the report highlighted several shortcomings of the quality of death in Hong Kong (including the lack of compulsory education on the subject in undergraduate medical training, the lack of legal standing of do-not-resuscitate, and limited understanding of the general public on the concept of palliative care), it only scratched the surface of the issues at hand.

While there is no overarching policy related to EOL care in Hong Kong, EOL care services are provided by both social and health care sectors. In the social care sector, non-governmental organizations (NGOs) provide much of the community care and support for older persons in Hong Kong, either independently or via funding from the Government’s Social Welfare Department. A major service provided is residential care services, and they can be roughly categorized into 1) Residential Care Homes for the Elderly (RCHEs) and 2) nursing homes, which require a licensing for meeting higher level criteria. RCHEs refer to any premises in which the care of persons is carried out for reward or other financial considerations [16]. In effect, many RCHEs are not equipped or prepared to deliver quality EOL care, and many frail older adults stay at RCHEs until death becomes imminent, and that is when they are usually sent to the public hospitals via ambulance for dying. Besides residential care, other community services may include but are not limited to community services at neighborhood elderly centers and day care centers, home care support, community EOL care, education, training workshops for caregivers, and bereavement care. Nevertheless, these services represent piecemeal efforts from various organizations that are not coordinated, and therefore, may overlap or leave gaps unattended.

On the other hand, in the health care sector, adult palliative care service was first started in 1982; since then, there was a steady growth of palliative care, especially after the establishment of the Hospital Authority (HA) to manage all public hospitals in Hong Kong in 1990. Currently, there are 16 out of 43 HA public hospitals that provide palliative care services, which are led by palliative care specialists either in the medicine or oncology department [17]. These services include physical palliative care with emphasis on symptom control, psychological counseling and support to patients and their family, assistance over social difficulties, and spiritual support, and they are provided in the forms of inpatient and consultative palliative care, ambulatory palliative care, community/home palliative care, bereavement service, and other supportive services. Nevertheless, there is inadequate coordination between the social and medical sectors for EOL care. As an effort to cope with this issue, in 2015, the HA started piloting an EOL care program in designated clusters in collaboration with selected RCHEs to train social care staff to identify suitable residents and/or their family to initiate EOL care discussions (including advance care planning, which is “a process of communication among patients, their healthcare providers, their families, and important others regarding the kind of care that will be considered appropriate when the patient cannot make decision” [18]) and to arrange coordinated admission from RCHE to different HA departments including accidental and emergency (A&E) and in-patient departments. Lastly, outside of the HA public healthcare sector, EOL care services in the private healthcare sectors are largely underdeveloped [19]. These services are mainly fee-for-service private businesses that provide discretionary case support to individual clients, while private hospitals generally do not provide EOL care to their patients.

Therefore, research that systematically investigates the service gaps, issues, and barriers of EOL care in Hong Kong from a more holistic approach, centering on the micro-macro linkages and dynamism, is much needed. In order to devise effective recommendations for improving the quality of death and EOL care in Hong Kong, we need to delve further into identifying the major existing gaps and issues of the EOL care and understand the reasons and contexts behind them. This is the objective of the present study. Findings can highlight the multi-level gaps and issues of EOL care in Hong Kong, and shed light on how best to design more meticulous and comprehensive policy interventions that will likely have a more sustainable and instrumental impact on facilitating person-centered EOL care.

## 2. Materials and Methods

### 2.1. Study Design

We used a multi-method qualitative study design for data collection. Focus groups and in-depth interviews with key informants and stakeholders and longitudinal case studies with patients and families were conducted to collect our qualitative data. Ethical approval for this study was granted by the Survey and Behavioral Research Ethics Committee of the Chinese University of Hong Kong as well as all the HA Cluster Research Ethics Committees that oversee research studies conducted in the seven hospital clusters in Hong Kong (NTEC/CREC 2015.359; HKEC-2016-18; UW 16-087; KC/KE-16-0030/ER-3; NTWC/CREC/16026; KW/EX-16-096(100-02)).

### 2.2. Sampling and Data Collection

We purposively sampled our subjects based on a detailed mapping of the landscape of services supporting individuals who were approaching the end of life and arrangements after death. The subjects included individuals who were experiencing EOL care, their families and caretakers, policy makers, services providers in the health and social care sectors, lawyers, journalist, and other stakeholders. Multiple data collection methods were involved, including in-depth interviews in both individual and group forms, focus group discussions, and longitudinal case studies. Details of data collection are laid out below.

A semi-structured interview guide and focus group discussion guide were used for the discussion sessions, which were conducted in Cantonese. Based on literature review, the interview guides were formulated by experienced qualitative researchers in our team to comprise open-ended questions and probes for elaborations. They underwent rounds of revisions and were formatted according to the target interviewees under the same framework of study protocol. The objectives of the research were explained by the discussion at the beginning of the session, and participants’ consents were obtained prior to all data collection activities. An experienced researcher acted as the moderator of the discussion, while other research team members would be present as the note-takers. Focus group discussions and case studies were recorded and transcribed verbatim. For in-depth interviews, hand-written notes were taken instead of recording to respect participants’ preferences to ensure anonymity.

#### 2.2.1. In-Depth Interviews

We identified key informants and stakeholders in the service sector and collected detailed information on provision of services in group and/or individual interviews. Names and contacts of participants were identified on the internet and through personal networks of the research team and other participants. Participants in these interviews covered a wide range of services spanning across medical, business, social, and legal aspects of EOL care.

#### 2.2.2. Focus Groups with Health and Social Care Providers

To further explore key themes emerged from the in-depth interviews, focus groups were conducted with service providers in the health and social care sectors. Participants from the health sectors included allied health professionals (AHPs) from three hospitals representing the major geographic regions in the city (HK Island, New Territories, and Kowloon) and medical staff from EOL care programs in the public sector. Participants were contacted through connections of key informants in the in-depth interviews. On the other hand, those from the social care sectors included frontline and managerial staff from NGOs in the community. They were identified by the Hong Kong Council of Social Service, which was the federation of non-governmental social service agencies and had a database covering 80% of all NGOs in Hong Kong.

#### 2.2.3. Longitudinal Case Studies

In order to understand the experiences with EOL care including those provided at home and in EOL care programs in the public sector, dyads of patient-and-family caregivers were recruited for case studies. Participants were identified by medical staff in EOL care programs and referred to the research team for recruitment. The patients were diagnosed to be either terminally ill or suffering from life-limiting conditions. Each dyad was followed up for four months in total. The research team would try to contact the dyad every month after baseline interview to get an update on the participant’s experiences with the program, including thoughts and beliefs relating to the advance care planning (ACP), as well as their general physical and mental well-being.

### 2.3. Data Analysis

**Figure 1:**
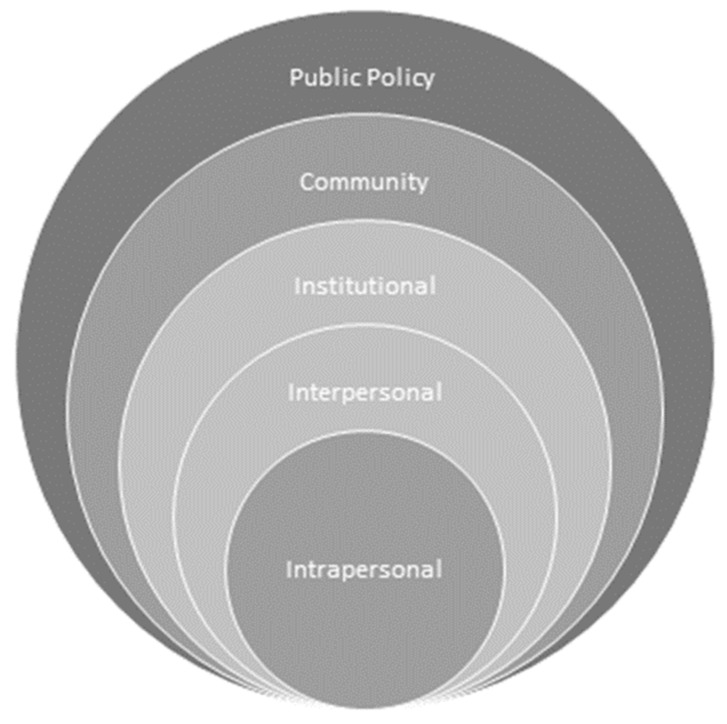
Socioecological framework (adapted from McLeory et al. 1988 [20]).

A deductive thematic analysis approach was used to identify the service gaps, issues, and barriers of the current EOL care for terminal illness and life-limiting conditions among older persons in Hong Kong. A socioecological framework commonly used for studying behaviors relating to health promotion interventions was adapted for organization and interpretations of the data collected. The McLeory et al. [20] framework focuses attention on intrapersonal factors (individual characteristics such as knowledge, attitudes, behavior, self-concept, skills, etc.), interpersonal factors (formal and informal social network and support systems), institutional factors (social institutions with organizational characteristics, and formal and informal rules and regulations for operation), community factors (relationships among organizations, institutions, and informal networks within defined boundaries), and public policy-level factors (laws and policies). The framework is illustrated schematically in Figure 1.

Three co-authors independently conducted line-by-line coding on a series of transcripts and notes to identify preliminary categories and met to discuss and decide on the analytic direction for the study based on research questions. The subsequent coding was completed by one author with regular meetings with the other two authors to refine coding schema until no new information can be identified from the data. Hence, data saturation was reached. Rigor in data analysis rested on strategies including independent coding by research team members and data triangulation by the different data collection methods.

## 3. Results

For the in-depth interviews, we conducted a total of seven group interviews with 42 key informants who served as hospital chief executives, chief of service, geriatricians, nursing consultants or managers in the seven hospital clusters in Hong Kong. Twenty-six of them were male. In addition, 31 interviews were conducted with 33 stakeholders providing EOL-related services with 29 of the interviews completed one-on-one. Twenty-seven of them were male. All participants were ethnic Chinese. Each interview was generally 60 to 90 min in length. For the focus group interviews with the health and social care providers, a total of 29 focus group discussion sessions were conducted: six with AHPs (*n* = 42), 16 with various NGOs (*n* = 75), and seven with medical staff working in EOL care programs in public hospitals (*n* = 17). All participants were ethnic Chinese. AHPs included physiotherapists, occupational therapists, dieticians, podiatrists, speech therapists, clinical psychologists, radiologists, and medical social workers. Self-reported work experience after obtaining professional qualification ranged from >16 years (*n* = 31), 11–15 years (*n* = 7), 6-10 years (*n* = 2), and 3–5 years (*n* = 2). Of the AHPs interviewed, 23 were male and 19 females. The 75 participants from the NGOs included frontline staff including care workers, health workers, nurses, social workers, physiotherapists, and managerial staff. 65 of the 75 were female. Medical staff included three doctors and 14 nurses. Each focus group had three to nine participants and lasted 90 to 180 min long. For the longitudinal case studies, 14 dyads of patient-and-family caregivers were recruited. Among the 14 cases, 13 had participated in ACP discussions, and 12 were enrolled in the EOL care program run by three hospitals in Hong Kong. One case had knowledge about the EOL care program provided by a hospital located in another cluster but had not yet participated in ACP. A total of 12 EOL care patients were females and two were males, and their age ranged from 74 years to 102 years. The majority of them (*n* = 11) were receiving residential care at old age homes, and three were living with their adult children and receiving home care at the time of the interview. Because the patients in the participating dyads did not have the cognitive capacities to understand ACPs or EOL care, interviews were completed with caregivers, including the relatives of the patients i.e., spouse (*n* = 2), adult children (*n* = 11), or godson who had a close relationship with the patient (*n* = 1). The informal caregivers were between ages 36 and 80 years. In total, 204 people were interviewed.

In this study, we focused on factors that affected the quality of as well as services gaps in current EOL care. Through the lens of the McLeory et al. socioecological model, EOL service gaps and issues were regarded as embedded in various nested, hierarchical levels. In the following analysis, these factors, gaps, and issues are unhitched from each level and studied in detail.

### 3.1. Intrapersonal and Interpersonal Factors

#### 3.1.1. General Reluctance and Fear of the Topics of Death and Dying

Although older people were generally more willing and likely to engage in conversations about death and EOL care compared to their younger counterparts, due to the inadequate knowledge and awareness of the concepts ‘aging in place’ and ‘dying in place’ in Hong Kong, there was a general reluctance from patients and their families to think about death and the dying process. Such a reluctance in turn made it challenging for healthcare professionals to have discussions with patients and their family members about the issues. As a result, the general lack of understanding about life-and-death and EOL care issues in all sectors of Hong Kong seemed to contribute to the cyclical self-reinforcing association between death being a cultural taboo and inadequate discussion and education on death and EOL care issues.

The problem was compounded by uncertainty in prognostication, especially for non-cancer patients. As an old woman’s daughter described:

“*Mom never mentioned these things, and she didn’t like it when we mentioned things that were not good… like death and such. She didn’t like hearing about it. All she said was, ‘I would have a long life and live to one hundred’… So I got the impression that she didn’t think she would die one day.*”

(TMH-02-daughter)

#### 3.1.2. Filial Piety and Guilt towards Dying Family Members

The role family plays in terms of death and EOL care issues is significant in Hong Kong. For instance, there seemed to be a common notion that ‘everything must be done’ to save the patients, in accordance of the traditional belief and practice of filial piety. The family tended to try the utmost to maintain the patients’ lives even though it may not be in line with the patients’ own preferences. Family members of patients, especially next of kin, expressed that they might tend to experience feelings of guilt if they felt that they had not cared enough for the dying person during their EOL. Consensus could be hard to reach among siblings:

“*…Our plan aims to have people die in dignity and not to resuscitate. So he (the brother) thinks if we don’t resuscitate, isn’t that equivalent to watching her (mom) die? If we don’t give her (mom) the feeding tube, isn’t that equivalent to watching her starve to death? …*”

(HKEC-02-daughter)

We also found that many stakeholders wondered whether family members of patients would still make the same care/treatment decisions if they were to be well-informed about the (lack of) effectiveness and futility of the treatments and potential detrimental effects of aggressive or invasive treatments, such as cardiopulmonary resuscitation. However, from the patient families’ point of view, they were not informed with the full picture of the treatments:

“*… The doctors actually explained in detail, but they won’t really tell you the whole truth regarding resuscitation… he (the doctor) won’t, he won’t say in his position, like things would go down like that on the bed, or if he performed resuscitation, then this would happen. He would say there would be some pain, but he would not say the bones could be all shattered. So things like that, I hear from my sister (she works in healthcare).*”

(KEC-02-daughters)

Moreover, there was generally inadequate discussion and dialogue between the patients and their family members to understand the patients’ autonomous wishes, which echoes with the general reluctance and fear of the topics of death and dying at the intrapersonal level. Some mentioned their wishes in transit like commenting on TV dramas showing situation in which someone is dying:

“*The three of us sisters were discussing what to do with mom basing on what she had said a few times when watching dying scenes on TV that she did not want any tubes, so we tried to respect her wishes… but we still fought over whether to give her feeding tubes before because all mom said was ‘no tubes’ but did not say what types of tubes…*”

(KEC-02 - daughter)

### 3.2. Institutional Factors

#### 3.2.1. Inadequate Knowledge, Training, and Resources of EOL and Palliative Care in the Healthcare Sector

In Hong Kong, the medical culture and mindset favor medical intervention of curative nature over palliative care. Professionals who are unfamiliar with palliative care principles often perceive palliative or EOL care as synonymous with giving up hope, rather than with placing patient respect and autonomy at the center of care. Therefore, treatments may be aggressive or intrusive. Challenges to providing quality care stem from inadequate basic training and experience, ethical concerns over when palliative care is appropriate (such as withholding treatments), feeling unfamiliar and unprepared with EOL care practices for those in the last weeks or days of life, and fear of legal liability in withholding treatments. There was a consensus that the university undergraduate medical curriculum in Hong Kong focused on curing patients until the very last moment but lacked emphasis on facilitating a good dying process for patients. Sometimes, even if the patient’s family did not want to overuse interventions, the doctor would insist:

“*Yes, I told him (the doctor) I did not want my mom to suffer. I did not want to put a feeding tube in her, then he said ‘Did you mean you wanted to watch her die? Because she couldn’t eat on her own and you would not put a tube in her, then she would die.’ Just like that. It’s true.*”

(HKEC-02-daughter)

EOL care in Hong Kong is often delivered by professionals that specialize in palliative or geriatric medicine. However, the number of palliative care specialists is currently small, despite increasing demand. There were only about 40 doctors, 300 nurses and 60 allied health professionals who acted as palliative care service providers in Hong Kong. The coverage was also narrow. Palliative care was received by 64% and 44% of cancer and end-stage renal patients according to a study published in 2016 [21], respectively. Not all EOL care patients were referred to receive palliative care in a timely manner; for example, despite a high prevalence of dementia among the older persons, palliative care services were not routinely provided to patients with progressive neurodegenerative disorders who were approaching their EOL, although some might receive palliative care in the very last weeks or days of life. On the other hand, for EOL care patients admitted to the hospitals, some might be attended by non-palliative care specialists without adequate palliative and EOL care training, and who might not engage or consult the palliative care team until the very late stage. In Hong Kong, since any patients (including EOL care patients) are mostly admitted to public hospitals via the A&E Department, those who are in need of EOL and palliative care would be attended by medical practitioners in the A&E settings, where palliative care would not be administered. As expressed by a healthcare provider, there was insufficient understanding, coordination, and communication in the A&E Departments to handle EOL care patients in general (*Ind-11-doctor*).

#### 3.2.2. Inadequate and Inappropriate Transportation for EOL Care Patients

It is common for persons in the end stage of life frequent hospitals as their health deteriorates because caregivers at home or the old age home may not have the needed knowledge or skills to manage worsened symptoms. The main mode to transfer patients from the community to hospital is through ambulances to the A&E Department. In effect, although many EOL care patients may not need acute care services, they still need to go through this process before being transferred to extended or sub-acute care facilities. Transportation to healthcare facilities poses significant inconvenience to EOL care patients with functional impairment or mobility issues. Non-emergency ambulatory transfer (NEAT) services do exist for mobility-disabled patients who are unable to take public transportation; however, capacity is limited. Booking is needed “60 days in advance and they are not always available” as reported by a caregiver of an EOL care patient (SH-01-daughter), and eligibility is usually restricted to patients not escorted by friends or relatives who live alone, are bedbound, are wheelchair-bound without direct elevator access, require constant oxygen supply, or have mental or sensory impairment. Hospitals are usually hectic, congested, risky for contracting infectious diseases, and are not necessarily the ideal setting to facilitate EOL care and peaceful dying. Privacy is also limited, and opportunity for relatives and loved ones to spend time with patients is limited by visiting hours and the acute ward environment.

As an effort to improve the quality of services for EOL care patients and their family members, the HA started implementing EOL care programs across some of its hospitals since 2015. The program piloted a new service pathway for its members to arrange admissions with EOL wards in the hospital directly depending on space availability for symptom management. This saved the patients and their families the long hours of waiting at the A&E departments and increased continuity of care by avoiding unnecessary medical investigations for patients. The EOL care programs also allowed the use of NEAT services during office hours. If NEAT was not available, they would need to arrange their own transportation or call an ambulance and go through A&E departments. As the majority of ambulances are under the purview of the Fire Services Department, they are bounded by the Fire Services Ordinance which mandates resuscitation if needed. This ambulance transfer method may bring the risk of possible futile resuscitation and other intrusive treatments against patient wishes. As a manager of the EOL program lamented:

“*…Because the paramedics have their own rules that we can’t interfere, and this is something that remains unresolved after many years…*”

(FG-KT-Manager)

#### 3.2.3. Inadequate Knowledge, Training, and Resources of EOL Care in the Social Care Sector

Our findings show that there was a general understanding that old age homes (including RCHEs and even the higher-level nursing homes in which death is legally permissible) would not administer EOL care, and their staff members were reluctant to engage in and take responsibility for care during the last days of life, because they were accustomed to the idea that EOL care and dying should happen in the hospitals. Inadequate staffing, support, and resources added to the belief that old age homes were not the appropriate settings to provide care for the last days of life, and resulted in the practice of rushing dying patients to hospitals. There was also a lack of essential equipment such as catheters, needles, and dressings, in addition to wheelchairs and oxygen supply and equipment for basic care and symptom control (e.g., oral suction, IV drip/syringe pump, etc.) in the old age homes. The lack of availability, storage, and dispensing capacity of prescription drugs in old age homes may also affect timely relief of symptoms. As a result of the inadequate knowledge, training, and resources to handle complex cases in the social care sector, EOL care patients who were residents of old age homes may not receive the appropriate care and might experience frequent transfer between the old age homes and hospitals:

“*… but how about the quality of the services provided by these caregivers (in the old age home)? Only one or two were actually certified. They are actually all migrant workers from the Mainland. I don’t know if they had training in the Mainland, but many were new to the field and very green… Is there someone from this home who gave them training? I don’t think so…. The old age homes run by the government had all their staff certified. Regardless of experience, at least the staff went through training…*”

(HKEC-02-daughter)

#### 3.2.4. Inadequate Medical-Social Interface and Coordination

EOL care and services were inadequately coordinated between medical and social care sectors and did not enable a continuum of care when patients transited between the two sectors, despite some ad-hoc EOL care programs and pilot programs at the HA. The inadequacies in coordination included referral, transfer, information sharing, and access that ensured delivery of timely, appropriate, and continuous care in the transition. There were also shortcomings in the mechanisms and systems that enabled multi-disciplinary care. For instance, there was a general consensus in the social sector that it was difficult to access medical records of patients so there was limited knowledge of pre-discharge hospital treatments, follow-up care plans, or choices for EOL care. Some key informants from the social sector also expressed that sometimes they did not understand the rationale and appropriateness of some of the treatments received in the hospital settings, and even questioned whether some of the medical care received were consistent with the principle of good EOL care:

“*The doctors at A&E departments do not know some of these EOL cases and perform unnecessary procedures (on our cases)… this could mean unnecessary suffering for our cases.*”

(FG GP7-PLK-Manager)

On the other hand, some medical staff felt that some medical records were too difficult and complicated for non-medical professionals to understand, and the information of which may even be unnecessary for long-term care in the social sector. This lack of common understanding might lead to distrust and confusion between the two sectors. However, both sectors believed that selected (not all) record sharing may help, but there was no consensus as to the amount and type of information to be shared.

#### 3.2.5. Inadequate Awareness of Advance Care Planning and Advance Directive in the Public Healthcare and Social Care Systems

Advance care planning, being an integral part of EOL care [18], can document both medical (usually including do-not-attempt-cardiopulmonary-resuscitation directive and life-sustaining treatments) and non-medical (including personal, caregiver, financial, and legal implications) care preferences of patients’ wishes, taking socio-cultural considerations into account. It has been found that ACP improved completion of advance directive (AD) that formally documents the patient’s medical preferences made in advance when they are mentally capacitated, improved patient and family outcomes, decreased life-sustaining treatments, increased palliative care, and increased the concordance between patient wishes and the care received [2,22,23,24]. Despite its potential benefits and the updated HA Guidelines on Life-sustaining Treatment in the Terminally Ill published in 2015 which offered an approach for ACP suitable for the local context [25], we found that ACP was not routinely promoted or universally recognized across all parts of the health and social care system in Hong Kong. It is only in recent years that several clusters of the HA public hospitals, in collaboration with selected old age homes, implemented a pilot EOL care program that made use of ACP. Its use had also seen an increase among non-cancer geriatric patients outside of the piloting clusters. Outside of the hospital settings, and apart from the old age home participants of the above-mentioned pilot EOL care program, ACPs were also infrequently made in most of the social care settings. Old age home staff, particularly the non-medical ones, were also generally unfamiliar with the concepts and guidelines for ACP; as a result, there was uncertainty and fear over following ACPs and determining liability for care.

“*We have a very big responsibility… It’s very hard to judge. If the family had a problem with it, they would sue you for negligence. It could be a legal issue.*”

(FG-LP Staff at old age home)

Even when there was an ACP made, we also found that ACP conversations were usually started too late when the patient had already started losing his/her mental capacity due to declining condition. As of the end of 2017 when we finished our data collection, there was no formal guideline on ACP issued by the HA.

On a related note, the HA had issued the Guidance for HA Clinicians on Advance Directives in Adults in 2014 [26]; however, the uptake was relatively slow probably due to inadequate awareness and knowledge of ADs in the general public, uncertainties over the duration of AD validity and options to revoke a previous decision due to change of mind, reluctance among healthcare professionals to start difficult EOL care conversations, and concerns over the lack of legal protection for the healthcare providers who followed ADs. In fact, a doctor said in the interview that some fellow doctors strongly resisted the idea of EOL services, and views among families were usually polarized (Ind-24-doctor). In the same year, HA also issued its guidelines on “Do-Not-Attempt-Cardiopulmonary-Resuscitation (DNACPR)” [27], within which a standard form was developed to extend DNACPR directives from the original hospitalized patients to those non-hospitalized and discharged. Nevertheless, the form was not widely understood and acknowledged outside of the HA hospitals, including the old age homes, and more importantly the Fire Services Department which run the ambulatory services in Hong Kong:

“*I don’t think the ambulance knows about the EOL programs and the ACPs… They (the paramedics) argued with me, like for a while, and said ‘No way. Of course we need to go back to the Prince of Wales Hospital (acute hospital). How can I take you to the Shatin Hospital (rehabilitation hospital)?’ I said, ‘but that’s what the doctor told me to tell you.*”

(SH-01-daughter)

This problem was compounded with limited or ineffective mechanisms to identify patients with ADs in other sectors which could not readily access the HA clinical management system.

#### 3.2.6. Inadequate and Inappropriate Emphasis, Coverage, and Provision of Bereavement and Decedent Care

There was inadequate awareness, and hence low utilization, of bereavement care despite these services being available at affordable costs in Hong Kong. Even though the HA provided bereavement services to the family after death of the patients, it has been identified in our study that medical records in the information system were archived after a period following death of the patients, which did not allow further inputs and follow-up, and might act as a barrier to continuous bereavement services.

Moreover, the Food and Environmental Hygiene Department of the Hong Kong Government is the primary unit to manage the after-death arrangements of the deceased body. These services include registration of death, application and arrangements for cremation/burial, licensing of coffin shops (i.e., business or trade operated by a person who undertakes all or any duties connected with the cremation or burial of human cadavers), as well as the scattering of cremated ashes at sea. However, a caregiver in the interview recalled that some morticians from the public mortuary “*placed [her] father’s body on the floor for the family to see*” (*SH-03-daughter*), for which she felt very upset.

### 3.3. Community Factors

#### 3.3.1. Service Gaps and Overlaps for EOL Care in the Community

Due to poor coordination among the many different care providers and that the services had been developed over time in a piecemeal fashion, there were both service gaps and overlaps at the same time. In other words, some patients might receive overlapping services, while some others might receive inadequate or even no services. Despite individual efforts from different programs to avoid overlapping of services, there was no consistent, patient-centered approach that oversaw the delivery of EOL care to each individual.

#### 3.3.2. Insufficient Resources for Effective EOL Care in the Community

As in residential care settings, there was also a lack of essential equipment for basic care and symptom control in the community to support “aging in place.” The lack of space at patients’ home was also a major issue for patients who need assistance for mobility and daily activities. The lack of availability and storage of prescription drugs at patients’ homes might also affect timely relief of symptoms. If these EOL care patients were not attended by palliative care teams from the hospital, it would be unlikely that they would receive any palliative community care, and many of them were principally cared for by informal caregivers with no specific training in EOL care.

#### 3.3.3. Insufficient Support for Caregivers of Home-Dwelling Patients

There was generally low awareness of how to provide care for patients approaching EOL or where and how to access community EOL services among informal caregivers in the community. There was also an inadequate socio-cultural environment to support caring for EOL care patients in the community. While there was paid maternity leave in Hong Kong, there was no paid EOL caregiving or bereavement leave for the caregivers. Since most caregivers also belonged to the working population, they would not be readily available to care for their EOL patients; as a result, many families in Hong Kong relied on foreign domestic workers for support and care. Nevertheless, there was no requirement for foreign domestic workers to be trained for EOL care; hence, support might not be sufficient or even appropriate. Some palliative care units did provide training to informal or paid caregivers (including domestic workers); however, they were only limited to patients under the care of these palliative care units, of which the coverage was very limited. There were also other caregiver support services provided by the Social Welfare Department in the community; however, they aimed at training and supporting caregivers for general care of older patients, but not specifically EOL care. Therefore, the feeling of being neglected and helpless was pervasive among the caretakers:

“*I think what is even more important is the support from the society/community….Her (the patient’s) conditions keep changing and there should be more support for us (caregivers for home-dwelling patients), like training courses or briefings and more complete courses for patients entering different stages… what facilities we need to add at home… especially when things are changing quickly and you are trying to deal with the situation, but you are also going to work at the same time…Ya I think that’s important.*”

(KEC-02– daughters)

### 3.4. Public Policy-Level Factors

Finally, many of the gaps and issues identified in the more downstream levels were present due to more macro-level, upstream factors pertaining to public policies and laws. While there were many EOL care services in Hong Kong, as described in the Introduction section, an overarching policy related to EOL care necessary to drive forward changes was missing.

#### 3.4.1. Inadequate Legal Definition for Mental Incapacity

In Hong Kong, the legal definition for ‘mentally incapacitated person’ was inadequate. The Mental Health Ordinance (Cap. 136) [28] was originally enacted for people with mental illness or who were mentally compromised or handicapped. However, there was no specific provision for persons who were mentally incapacitated from other conditions such as those suffering from a persistent vegetative state, or in an irreversible coma and persons with dementia. Despite the Law Reform Commission (LRC)’s 2006 proposal [29] for new definitions of mental incapacity by bringing comatose and vegetative persons within the protection of the existing legal framework, no necessary changes were made. As a result, the burden of deciding whether patients were mentally capable lied with the clinicians because the legal definitions were not specific and provisions were not made for conditions specific to aging. For instance, mental incapacity might fluctuate such as with dementia, but there was no guidance for clinicians on how to handle this in terms of identifying mental incapacity. This also had direct effect on when the directives made in the ADs took effect.

#### 3.4.2. Lack of Legislation for Advance Directives and Potential Conflicts

As mentioned earlier, in 2014, the HA provided standardized full and short versions of ADs to all HA public hospitals in Hong Kong with its ‘Guidance for HA Clinicians on Advance Directives in Adults’ [26]. Yet, AD was still not widely recognized either in the general public or in the medical sector. Moreover, AD was not legislated, but only governed under the common law framework. In other words, a valid AD would be legally recognized unless challenged on the grounds such as incapacity or undue influence under the common law framework. However, uncertainties do remain regarding ADs under the common law. Specifically, decisions made in the ADs, albeit valid ones, could be superseded in cases of conflict with other statutory provisions, as illustrated below:

AD vs. ‘best interest principle’: There might be potential conflict between an AD made in advance for someone who later became mentally incapacitated and the obligation for practitioners to carry out treatment in the ‘best interest’ of the patients. The Mental Health Ordinance (Cap. 136) Section 59ZF in Hong Kong states:

“*Where a registered medical practitioner … considers that treatment is necessary and is in the best interests of the mentally incapacitated person, then he may carry out that treatment without the consent of the mentally incapacitated person or that person’s guardian (if any) accordingly.*”

In effect, without legislation instructing clinicians to follow ACP wishes or ADs, clinicians may technically select treatment according to what they believe to be in the best interest of a patient, even when it may not agree with the patient’s autonomous wishes expressed in the AD.

AD vs. Fire Service Ordinance: There might be potential conflict between wishes expressed in ADs and the Fire Services Ordinance (Cap. 95) obligation to resuscitate or sustain life [30]. Under the current interpretation of the law, the Fire Service Department (FSD) was often obliged to perform resuscitation. As mentioned, the HA had implemented a DNACPR system for hospitalized patients and expanded the scope to non-hospitalized patients with an AD or ACP in 2014. However, the FSD’s non-participation in this guideline meant FSD ambulance staff might still resuscitate non-hospitalized patients even when the effort was medically futile and against their expressed wishes.

AD vs. Enduring Powers of Attorney (EPA): Appointing ‘powers of attorney’ is a legal instrument used to delegate legal authority to another person (i.e., the attorney) when a patient (i.e., the donor) subsequently becomes mentally incompetent. The Powers of Attorney Ordinance (Cap. 31) in Hong Kong only allowed the appointed attorney to handle property and financial matters of the donor before and after he/she becomes mentally incapacitated [31]. However, patients may not wish to formally document care preferences using ADs because of the uncertainty in prognosis or the difficulty in planning for one’s own death. In such cases, being able to appoint trusted attorneys to act on one’s behalf may bring comfort to patients and their family members. While safeguards and caution are important, excluding life-sustaining treatment from EPA decisions does not reflect and address the reality of many situations where patients lack mental capacity, have not made ADs, and important treatment decisions on life-sustaining treatment must be made. Therefore, if EPA is to be extended to include decisions on life-sustaining treatments, and if AD is to be legislated, there might be potential conflict between treatment wishes expressed in an AD and the right for appointed attorneys to make decisions for patients who were not mentally competent. There was no legislation stating whether ADs or EPAs should take precedence in cases of conflict.

#### 3.4.3. Limited Power of Guardians in EOL Treatment Decisions

As stipulated in the Mental Health Ordinance (Cap. 136), guardians only have power to consent to medical treatments but not refuse [28]. However, decisions for patients in the last days of life often need to extend beyond consent for medical treatment to include refusal of futile or unnecessary treatment, tests, or interventions (*Ind-6-director*). Therefore changes, with appropriate safeguards, were needed within the legislation to ensure that appointed guardians can fully perform their role in acting on behalf of patients during their EOL.

#### 3.4.4. Inadequate EOL Care Capacity of Residential Care Homes for the Elderly

Most of the old age homes in Hong Kong were RCHEs, which were not required to have medical doctors on their staff per Residential Care Homes (Elderly Persons) Ordinance 2012, Section 22: Social Welfare Department Code of Practice [32]. RCHEs could purchase services from visiting medical officers, but they were not even required to visit the sites daily. EOL patients in these old age homes did not consistently receive adequate care, particularly out of office hours or when doctors were not on site, and staff of residential care homes would often resort to calling ambulances to send patients to the A&E departments triggering recurring hospitalizations.

“*When it’s midnight hours or 6 or 7 o’clock in the evening, I can’t arrange a doctor for our patients. The only thing I could do was to call an ambulance to send him/her to the A&E.*”

(FG-GP10_SA, Frontline Staff)

### 3.5. Summary of Main Findings

Using the socioecological framework, we hereby summarize in Table 1 the gaps and issues of EOL care in each of the ecological level for terminal illness and life-limiting conditions among older persons in Hong Kong.

## 4. Discussion

### 4.1. Interpretations and Policy Implications

In this study, we gave an overview of the multi-level service gaps and issues of EOL care in Hong Kong. In summary, although there were various practices and providers of EOL care in Hong Kong, as summarized in the Introduction section, there is no overarching policy framework related to EOL care that conceptualizes and clarifies patients’ and their family members’ holistic needs, and how they could be provided with a comprehensive system of care. An advantage of the present study is that it extended from previous works that focused primarily on perspectives from the care providers [33,34]. While there are consistent findings, the present study also encompasses opinions and observations of other major stakeholders of EOL care including the non-care providers. In addition, our findings are consistent with the ones found in another local study, where different samples were recruited [35]. In particular, that study found low priority on the policy agenda, lack of consistent funding to support care services, unfavorable culture for promoting EOL care (including denial of death, myths about filial piety, and strong belief in medical authority), EOL care being less alluring than biomedical sciences for career development of health professionals, undesirable environment for providing holistic care, as well as legal issues (including uncertainty about AD, limited powers of attorney and guardians, and duties of the ambulance men) being the gaps in the development of EOL care in Hong Kong. As a result, that study made a case to urge the government for formulating a policy framework to shape the EOL care and promote its development by implementing public health strategies, which is consistent with the finding of a lack of overarching policy framework related to EOL care in this study. This recommendation is also aligned with the evidence found in other countries, where support from governments and policymakers are essential for the development of EOL care [36,37,38,39,40].

The findings of this study have significant implications for the development of an overarching, and cross-sectoral EOL care policy in Hong Kong in order to drive forward change and institutionalize service integration. It is important to emphasize that any EOL care-related policy should not be just formulated to target care during the last months and days of life, but should be an integral part of a long-term care policy, since EOL care only happens to be the last phase of a longer continuum of care for patients with terminal illness or life-limiting condition, and therefore, cannot be separated from the earlier parts of the care. As shown in a previous study [41], the long-term care expenditure in Hong Kong, in parallel to secular changes in the elderly dependency ratio, was projected to monotonically increase from 2011 onwards; thus, now is a critical time to discuss and debate policy options that would address the burgeoning long-term care burden along with EOL care considerations.

As the findings of this study clearly demonstrate, many of the gaps and issues were politically, socially, and culturally specific. This is consistent with the literature, including the local study by Chan et al. [35] as well as the US Institute of Medicine’s “Dying in America. Improving quality and honoring individual preferences near the end of life” report [9]. Therefore, recommendations should need to be contextualized in a socio-culturally sensitive and appropriate manner, rather than merely adopted from international policies from other cultures and contexts. Nevertheless, common denominators of EOL care around the world was identified in a previous scoping review [42], and these can be taken for development of socio-culturally and locally appropriate policies and services. The objective is never to prescribe any particular form of future EOL care, but to establish a suite of solutions that uphold socio-culturally and locally specific values and principles for EOL care delivery that adequately and appropriately address the local issues.

Furthermore, it is important to ensure that the governmental policy for EOL care in Hong Kong is an evolving one that allows for necessary updates according to new practices and emerging evidence. For instance, knowledges, attitudes, and preferences of EOL care in Hong Kong were examined in a population-wide survey [43], the findings of which could be informative to policymaking. It is also important to note that the top-down institutional approach should not be the only force of change for the development of EOL care, as demonstrated previously [40,44]. Collaborations across different service sectors and organizations [39] as well as taking the public health approach on EOL care [45,46] are also integral to achieving equity in terms of access to and quality of EOL care. During and after the implementation of the present study, the HA had been piloting an EOL care program in several HA clusters in collaboration with selected old age homes to facilitate better coordinated and medico-socially-integrated care that included the ACP process, while other ad-hoc programs have been implemented by other NGOs and charitable organizations in collaboration with academic institutions. Evaluation of these pilot programs will inform future policies and measures. In addition, the HA published its Strategic Service Framework for Palliative Care on August 2017 [17] and a formal guideline on ACP on June 2019 [47] after the completion of our data collection. Moreover, the Government Food and Health Bureau completed its public consultation on legislative proposals on ADs in December 2019 [48]. All these new developments should be taken into account when formulating policies for future EOL care.

### 4.2. Limitations

The study design is qualitative in nature; in other words, our results did not have the statistical power to represent the views of the general public. Nevertheless, the purposively selected interviewees were major stakeholders and key informants of the field, and they tended to be better informed and might possess more insights into the issues at hand than the general public, many of whom might not even have any experience related to EOL care. The case studies of EOL patients and their caregivers should also give first-hand accounts of the authentic experiences related to EOL care in Hong Kong. The EOL care services and practices in Hong Kong are ever evolving, and our study did not capture the opinions on the latest progresses made and services developed after our data collection. Nevertheless, most of the gaps and issues found in our study have not been adequately resolved.

## 5. Conclusions

In light of the latest developments in EOL care, including the medical-social collaborative pilot EOL care program, a new strategic service framework for palliative care and a new formal guideline on ACP in the public healthcare sector, as well as the completion of the Government’s public consultancy on the legislative proposals on ADs, now is the critical time that offers the golden opportunity to capitalize on this momentum for formulating sustainable and crucial EOL care in Hong Kong. The rest of the world, especially those that are rapidly aging, can also take Hong Kong as a case study for the gaps and issues that they might face on the road to achieving good death for their people.

## Figures and Tables

**Table 1:** Gaps and issues of end-of-life (EOL) for terminal illness and life-limiting conditions among older persons in Hong Kong.

**Policy- and Legal-Level Gaps and Issues:**
A lack of overarching policy framework for EOL care
Ambiguity in the legal basis for mental incapacity and the legislative barriers for ADs (including potential conflicts of DNACPR or AD decisions with the duty to resuscitate required by the Fire Services Ordinance, ‘best interest’ principle, and appointed attorney decisions if it were to be extended beyond financial arrangements to include personal care on life-sustaining treatments);
Limited power of guardians in EOL treatment decisions; and
Inadequate capacity of residential care homes for the elderly to care for EOL patients due to limited legal requirements.
**Community-Level Gaps and Issues:**
A lack of consistent, patient-centered approach that avoids gaps and overlaps of services and oversees the delivery of care to each individual;
Insufficient resources and equipment to facilitate EOL care in the community; and
Insufficient support for caregivers of home-dwelling patients.
**Institutional-Level Gaps and Issues:**
Inadequate knowledge, training, and resources of EOL and palliative care in the healthcare sector;
Inadequate and inappropriate transportation for EOL care patients to extended care or sub-acute care facilities;
Inadequate knowledge, training, and resources of EOL care in the social care sector;
Inadequate medical-social interface and coordination;
Inadequate awareness of ACP and AD in the public healthcare and social care systems; and
Inadequate and inappropriate emphasis, coverage, and provision of bereavement and decedent care.
**Intrapersonal- and Interpersonal-Level Gaps and Issues:**
General reluctance and fear of the topics of death and dying; and
Interpretation of filial piety to resemble the practice of “doing everything possible” for dying family members

ACP—advance care planning; AD—advance directive.
